# A Simple and Selective Spectrophotometric Method for the Determination of Trace Gold in Real, Environmental, Biological, Geological and Soil Samples Using Bis (Salicylaldehyde) Orthophenylenediamine

**DOI:** 10.4137/aci.s977

**Published:** 2008-08-29

**Authors:** Rubina Soomro, M. Jamaluddin Ahmed, Najma Memon, Humaira Khan

**Affiliations:** 1 National Center of Excellence in Analytical Chemistry, University of Sindh, Jamshoro, Pakistan; 2 Department of Chemistry, University of Chittagong, Chittagong. 4331, Bangladesh

**Keywords:** spectrophotometry, gold determination, BSOPD, aqueous and micellar media, environmental, biological and soil samples

## Abstract

A simple high sensitive, selective, and rapid spectrophotometric method for the determination of trace gold based on the rapid reaction of gold(III) with bis(salicylaldehyde)orthophenylenediamine (BSOPD) in aqueous and micellar media has been developed. BSOPD reacts with gold(III) in slightly acidic solution to form a 1:1 brownish-yellow complex, which has an maximum absorption peak at 490 nm in both aqueous and micellar media. The most remarkable point of this method is that the molar absorptivities of the gold-BSOPD complex form in the presence of the nonionic TritonX-100 surfactant are almost a 10 times higher than the value observed in the aqueous solution, resulting in an increase in the sensitivity and selectivity of the method. The apparent molar absorptivities were found to be 2.3 × 10^4^ L mol^−1^ cm^−1^ and 2.5 × 10^5^ L mol^−1^ cm^−1^ in aqueous and micellar media, respectively. The reaction is instantaneous and the maximum absorbance was obtained after 10 min at 490 nm and remains constant for over 24 h at room temperature. The linear calibration graphs were obtained for 0.1–30 mg L^−1^ and 0.01–30 mg L^−1^ of gold(III) in aqueous and surfactant media, respectively. The interference from over 50 cations, anions and complexing agents has been studied at 1 mg L^−1^ of Au(III); most metal ions can be tolerated in considerable amounts in aqueous micellar solutions. The Sandell’s sensitivity, the limit of detection and relative standard deviation (n = 9) were found to be 5 ng cm^−2^, 1 ng mL^−1^ and 2%, respectively in aqueous micellar solutions. Its sensitivity and selectivity are remarkably higher than that of other reagents in the literature. The proposed method was successfully used in the determination of gold in several standard reference materials (alloys and steels), environmental water samples (potable and polluted), and biological samples (blood and urine), geological, soil and complex synthetic mixtures. The results obtained agree well with those samples analyzed by atomic absorption spectrophotometry (AAS).

## Introduction

The beauty and rarity of gold has led to its use in jewellary and coinage, and like a standard for monetary stems throughout the world. Gold is also one of most important noble metals due to its wide application in industry and economic activity. It has been used in medicine for quite some time.

A simple sensitive and selective method for determination of trace gold has been required. Sophisticated techniques, such as inductively coupled plasma mass spectrometry (ICP-MS) (Juvonen et al. 2002), inductively coupled plasma atomic emission spectrometry (ICP-AES) (Wu et al. 2004), electrochemical (Kavanoz et al. 2004), spectrophotometry (Zaijun et al. 2003), neutran activation analysis (Nat et al. 2004) and atomic absorption spectrophotometry (AAS) (Medved et al. 2004) have widely been applied to the determination of gold in various samples. Some factors such as initial cost of instrument, technical know-how, consumable and costly maintenance of technique restrict the wider applicability of these techniques, particularly in laboratories with limited budget in developing countries and for field work. A wide variety of spectrophotometric methods for determination of gold have been reported, each chromogenic system has its advantages and disadvantages with respect to sensitivity, selectivity and convenience (Alfonso and Gomez Ariza, 1981; Balcerzak et al. 2006; Chen et al. 2006; El-Zawawy et al. 1995; Fujita et al. 1999; Gangadharappa et al. 2004; Gao et al. 2005; Gowda et al. 1984; Hu et al. 2006; Koh et al. 1986; Matouskova et al. 1980; Melwanki et al. 2002; Mirza 1980; Ortuno et al. 1984; Pal 1999; Patell and Lieser, 1986; Zhao et al. 2006). A comparison of few selected procedures; their spectral characteristics and draw backs are enumerated in Table 1. The shiff-base reagents had widely been applied for the determination of noble metal ions, this type of reagent has higher sensitivity and high selectivity (Ahmed and Uddin, 2007). In the search for more sensitive shiff-base reagent, in this work, a new reagent bis(salicylaldehyde)orthophenylenediamine(BSOPD) was synthesized according to the method of (Salam and Chowdhury, 1997) and a color reaction of BSOPD with Au(III) in aqueous and micellar media was carefully studied.

The aim of present study is to develop a simpler direct spectrophotometric method for the trace determination of gold with BSOPD in aqueous solutions, and the presence of inexpensive nonionic micelles, such as polyoxyethylene octylphenyl ether (TX-100), in aqueous solutions.

## Experimental Section

### Instrumentation

A Perkin Elmer (Germany) (Model: Lambda-2) double-beam UV/VIS spectrophotometer with 1 cm matched quartz cells were used for all absorbance measurements. A pH-meter, WTW inolab (Germany) (Model: Level-1) combined electrodes were employed for measuring pH values. A Hitachi Ltd., Model 180–50, S.N. 5721–2 atomic absorption spectrophotometer with a deuterium lamp back ground corrector, equipped with graphite furnace GA-3, with Gold hollow cathode lamps of Hitachi, and a Hitachi Model 056 recorder was used for comparison of the results. The experimental conditions were: slit width, 1.3 nm; lamp current, 10.0 mA; wavelength, 242.8 nm; cuvette, cup; carrier gas (argon), 200 mL min^−1^; sample volume, 10 μL.

### Chemicals and reagents

All chemicals solvents used were of analytical reagent grade or the highest purity available. Doubly distilled de-ionized water, which is non-absorbent under ultraviolet radiation, was used throughout. Glass vessels were cleaned by soaking in acidified solutions of KMnO_4_ or K_2_Cr_2_O_7,_ followed by washing with concentrated HNO_3_ and rinsed several times with de—ionized water.

### Samples

Stock solutions and environmental water samples (1000 mL each) were kept in polypropylene bottles containing 1 mL of concentrated nitric acid. Biological fluids (blood and urine) were collected in polyethane bottles from affected persons (Jeweler’s who suffered from anemia, blood disorder, liver and kidney damage diseases). Immediately after collection, they were stored in a salt-ice mixture and later, at the laboratory, were kept at −20 °C (Ahmed and Mamun, 2001). More rigorous contamination control was used when one gold levels in the specimens were low.

#### Gold(III) standard solutions (5.08 × 10^−^^3^ M)

A 1000 mL stock solution (1000 mg mL^−1^) of gold was prepared by dissolving 1.0 g of gold (purity 99.999%) in aqua regia by warming, evaporating the solution to dryness, dissolving the residue in hydrochloric acid, evaporating the solution to half its volume, cooling and diluting with water to 1000 mL in calibrated flask (Zaijun et al. 2003). Working solutions were prepared by appropriate dilution of standard solution.

##### Bis(salicylaldehyde)orthophenylenediamine(BSOPD) (3.16 × 10^−3^ M)

The reagent was synthesized according to the method of (Salam and Chowdhury, 1997). The solution was prepared by dissolving the requisite amount of BSOPD in a known volume of double distilled ethanol (Merck, Darmstadt). More dilute solution of the reagent was prepared as required.

#### Polyoxyethylene octylphenyl ether (TX-100) (10%)

A 500 mL T-X100 solution was prepared by dissolving 50 mL of pure polyoxyethylene—octylphenyl ether (E. Merck Darmstadt, Germany) in 250–300 mL in doubly distilled de-ionized water, sonicated for 15 min and diluted up-to the mark with de-ionized water when it became transparent.

#### Aqueous ammonia solution

A 100 mL solution of aqueous ammonia was prepared by diluting 10 mL of concentrated NH_3_ (28%–30%) ACS grade with de-ionized water. The solution was stored in a polypropylene bottle.

#### EDTA solution

A 100 mL stock solution of EDTA (0.1% w/v) was prepared by dissolving 128 mg of ethylenediaminetetraacetic acid, disodium salt dehydrate (Merck, Darmstadt) in 100 mL de-ionized water.

#### Other solutions

Solutions of a large number of inorganic ions and complexing agents were prepared from their AnalaR grade, or equivalent grade, water-soluble salts. In the case of insoluble substances, a special dissolution method was adopted (Pal and Chowdhury, 1984).

## General Procedure

A series of standard solutions of a neutral aqueous solution containing 0.1–300 μg of gold in a 10 mL calibrated flask was mixed with 10–25-fold molar excess of the BSOPD solution (preferably 1.0 mL of 3.16 × 10^−4^ M) BSOPD reagent, 1–3.5 mL (preferably 2 mL) of 10% TX-100 solution, 0.5–1.2 mL (preferably 0.5 mL) of 4 M H_2_SO_4_. The mixture was diluted to the mark with de-ionized water. After standing for 10 min the absorbance was measured at 490 nm against a corresponding reagent blank. The gold content in an unknown samples (e.g. real, environmental, biological and soil samples) were determined using a concurrently prepared calibration graph.

## Results and Discussion

### Absorption spectra

**Figure 1.**
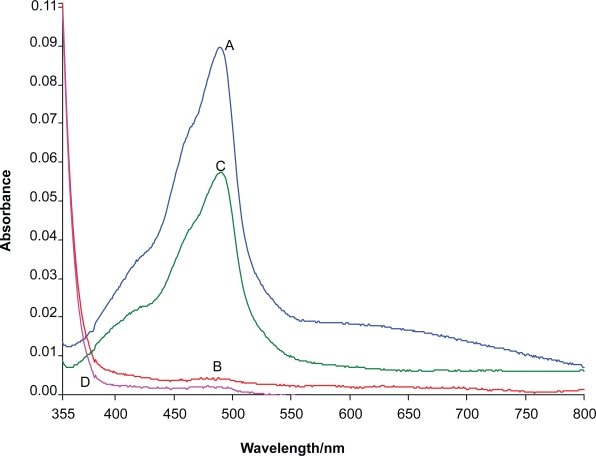
A and B absorption spectra of Au(III)-BSOPD system (1 mg L^−1^) and the reagent blank (λ_max_ = 490 nm) in micellar media, C and D absorption spectra of Au(III)-BSOPD system (1 mg L^−1^) and the reagent blank (λ_max_ = 490 nm) in aqueous solutions.

The absorption spectra of the Gold (III)-BSOPD system in a 4 M sulfuric acid medium were recorded using a spectrophotometer. The absorption spectra of the Gold(III)-BSOPD is a symmetric curve with the maximum absorbance at 490 nm and an average molar absorption coefficient of 2.3 × 10^4^ L mol^−1^ cm^−1^ and 2.5 × 10^5^ L mol^−1^ cm^−1^ in aqueous and micellar media, respectively Figure 1. The reagent blank exhibited negligible absorbance, despite having a wavelength in the same region. In all instances, measurements were made at 490 nm against a reagent blank.

#### Composition of the absorbance

**Figure 2.**
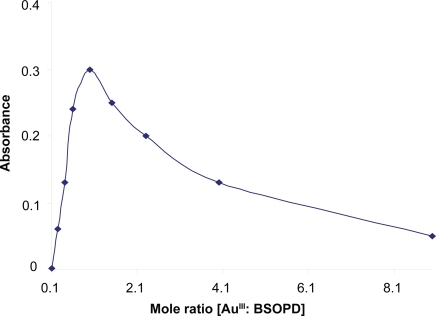
Composition of Au (III)-BSOPD complex by the Mole ratio method in micellar media.

Job’s method (Yoe and Jones, 1944) of continuous variation and the molar-ratio method were applied to ascertain the stoichiometric composition of the complex. Au-BSOPD (1:1) complex was indicated by the molar-ratio method (Fig. 2).

It was found that BSOPD has excellent analytical characteristics. In micellar medium (TX-100) BSOPD reacts with gold(III) to form a highly stable brown-yellow complex. Its apparent molar-absorptivity is 2.5 10^5^ L mol^−1^ cm^−1^, much higher than that of the other reagents in Table 1. Sensitivity can be measured in a very simple and rapid way and selectivity enhancement and low time-consuming as well as environmental and health safety due to avoiding the use of carcinogenic organic solvents are advantages of the method against previous extraction spectrophotometric method mentioned in Table 1. The method is far more sensitive, selective, non-extractive, simple and rapid than all of the existing spectrophotometric methods mentioned in Table 1. (Alfonso and Gomez Ariza, 1981; Balcerzak et al. 2006; Chen et al. 2006; El-Zawawy et al. 1995; Fujita et al. 1999; Gangadharappa et al. 2004; Gao et al. 2005; Gowda et al. 1984; Hu et al. 2006; Koh et al. 1986; Matouskova et al. 1980; Melwanki et al. 2002; Mirza, 1980; Ortuno et al. 1984; Pal, 1999; Patell and Lieser, 1986; Zhao et al. 2006). With suitable masking, the reaction can be made highly selective and reagent blank do not show any absorbance. The method is very reliable, and a concentration in the ngg^−1^ range in aqueous medium at room temperature (25 ± 5 °C)

### Optimization of some parameters on the absorbance

#### 

##### Effect of surfactant

Of the various surfactants {nonionic [polyoxyethylenedodecylether (Brij-35), polyoxyethylene sorbitan monopalmitate (Tween-40), polyoxyethylene sorbitan mono-oleate (Tween-80), TritonX-100]; cationic [cetyltrimethylammonium bromide (CTAB), cetylpyridinum chloride (CPC)]; and anionic [sodium dodecyl sulfate (SDS)]} studied, TritronX-100 was found to be the best surfactant for the system. In a 10% TritronX-100 medium, however, the maximum absorbance was observed; hence, a 10% TritronX-100 solution was used in the determination procedure.

Different volumes of 10% TritronX-100 were added to a fixed metal ion concentration, in 10 mL volumetric flask, and the absorbance was measured according to the standard procedure. It was observed that at 5 mg L^−1^ Au-chelate metal, 1–3.5 mL of 10% TritronX-100 produced a constant absorbance of the Au-chelate. Outside this range of surfactant (i.e. 10%–35%) the absorbance decreased. For all subsequent measurements, 2 mL of 10% TritronX-100 (i.e. 20%) was added.

The basic principle of spectrophotometric determination is Beer-Lambert Law (Beer’s) i.e. absorbance (A) is directly proportional to the concentration (C) of the analyte. The absorbance of the analyte is always measured against the corresponding reagent blank. When the sample (analyte) is diluted, in that case absorbance of reagent blank increased and does not obey the Beer’s Law.

#### Effect of acidity

Of the various acids (nitric, sulfuric, hydrochloric and phosphoric) studied, sulfuric acid was found to be the best acid for the system. The absorbance was at a maximum and constant when a 10 mL of solution (5 mg L^−1^) contained 0.5–1.2 mL of 4 M (2–4.8 M) sulfuric acid (or pH 0.83–1.2) at room temperature (25 ± 5 °C). Outside this range of acidity, 2–4.8 M (or pH 0.83–1.2) the absorbance decreased. For all subsequent measurements, 0.5 mL of 4 M (i.e. 2 M) sulfuric acid (or pH 1.14) was added.

#### Effect of time

**Figure 3.**
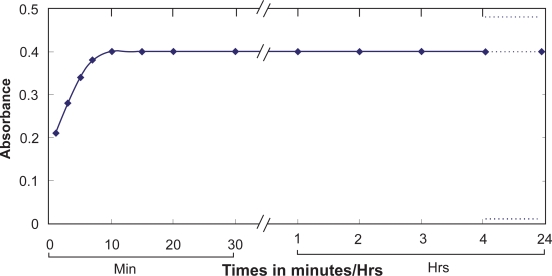
Effect of the time on the absorbance of Au(III)-BSOPD system.

The reaction is fast. Constant maximum absorbance was obtained just after 10 min of the dilution to volume at room temperature (25 ± 5 °C), and remained strictly unaltered for 24 h (Fig. 3).

#### Effect of temperature

The absorbance at different temperatures, 0–40 °C, of a 10 mL solution (5 mg L^−1^) was measured according to the standard procedure. The absorbance was found to be strictly unaltered throughout the temperature range of 10–40 °C. Therefore, all measurements were performed at room temperature (25 ± 5 °C).

#### Effect of the reagent concentration

**Figure 4.**
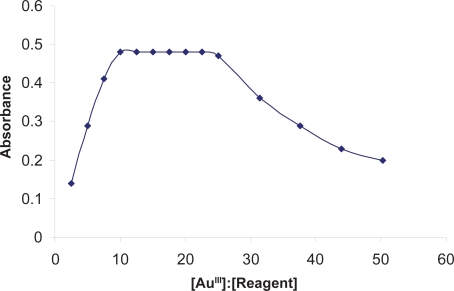
Effect of reagent [BSOPD: AU(III) molar concentration ratio] on the absorbance of Au(III)-BSOPD system in micellar media.

Different molar excesses of BSOPD were added to a fixed metal-ion concentration, and the absorbances were measured according to the general procedure. It was observed that at 0.5 mg L^−1^ Au-metal (optical path length, 1 cm), reagent molar ratios 1:10 and 1:25 produced a constant absorbance of the Au-chelate (Fig. 4). The effect of reagent at different concentration of Au^III^ (5 mg L^−1^) was also studied but similar effect was observed. For all subsequent measurements, 1.0 mL of 3.16 × 10^−3^ M BSOPD reagent was added.

### Analytical performance of the method

#### 

##### Calibration curve

**Figure 5.**
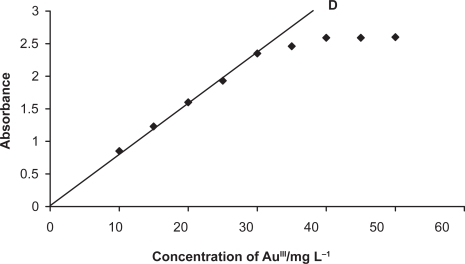
Calibration graph: D, 10–30 mg L^−1^ of Au (III).

The effect of metal concentration was studied over 0.01–100 mg L^−1^, distributed in four different sets (0.01–0.1, 0.1–1, 1–10, 10–100 mg L^−1^) for convenience of the measurement. The absorbance was linear for 0.1–30 mg L^−1^ and 0.01–30 mg L^−1^ of gold (III) in aqueous and surfactant media, respectively. From the slope of the calibration graph, the average molar absorption coefficient was found to be 2.3 × 10^4^ L mol^−1^ cm^−1^ and 2.5 × 10^5^ L mol^−1^ cm^−1^ in aqueous and micellar media, respectively. Of the four calibration graphs, the one showing the limit of the linearity range is given in Fig. 5; the next two were straight-line graphs passing through the origin (R^2^ = 0.9986). The selected analytical parameters obtained with the optimization experiments are summarized in Table 2.

##### Precision and accuracy

The precision of the present method was evaluated by determining different concentrations of gold (each analyzed at least five times). The relative standard deviation (n = 5) was 2.5%–0%, for 0.1–300 μg of Au^III^ in 10 mL, indicating that this method is highly precise and reproducible. The detection limit (Mitra, 2003) (3s of the blank) and Sandell’s sensitivity (Sandell, 1965) (concentration for 0.001 absorbance unit) for gold(III) were found to be 1 ng mL^−1^, 5 ng cm^−2^, respectively. The method was also tested by analyzing several synthetic mixtures containing gold(III) and diverse ions shown in Table 4. The results of the total gold in a number of real samples were in good agreement with the certified values in Table 5. The reliability of our Au-chelate procedure was tested by recovery studies. The average percentage recovery obtained for the addition of a gold(III) spike to some environmental water samples was quantitative, as shown in Table 6. The results of biological and geological analyses by the spectrophotometric method were in excellent agreement with those obtained by AAS (Table 7). Hence, the precision and accuracy of the method were excellent.

### Micellar Mechanism

BSOPD is an organic colorimetric reagent that provides basis of sensitive methods for the determination of a number of metal ions (Ahmed and Uddin, 2007). Gold ion combines with BSOPD to yield non-polar colored complex whose color differ significantly from BSOPD. This non-polar colored complex is generally extracted into organic solvents which is time—consuming and tedious. This problem has been overcome in recent time by introducing a hydrophobic micellar system generated by a surfactant. Micellar systems are convenient to use because they are optically transparent, readily available, and relatively non-toxic and stable (Tani et al. 1998). Nevertheless, the addition of surfactant at concentration above the critical micelle concentration (CMC) to an aqueous medium to form a micellar solution is the most commonly preferred procedure today. Micelles enhance the solubility of organic compounds in water by providing local non-polar environments. This phenomenon of micellar solubilization has been used in the development of many new methods and modification of existing methods of analysis (Paramauro et al. 1992). The use of micelle formation is promising for improving the analytical performance of the spectrophotometric procedure (Khan et al. 2007). Especially, the surfactants have been used to improve UV-visible spectrophotometric determination of metal ions with complexing agents (e.g. BSOPD, BBSOPD, etc). Generally the metal-chelate complexes formed (e.g. with shiff-base reagents) in the surfactant media (e.g. TX-100) are more stable than those formed in the absence of surfactant (e.g. aqueous media) (Ghazy et al. 2006).

### Effect of foreign Ions

The effect of over 50 cations, anions and complexing agents on the determination of only 1 mg L^−1^ of Au^III^ was studied. The criterion for interference (Ojeda et al. 1987) was an absorbance value varying by more than 5% from the expected value for Au^III^ alone. There was no interference from the following 1000 fold amount of acetate, chlorides, sulfate or oxalate; a 500-fold amount of EDTA or carbonate ; a 200-fold amount of tartrate, phosphate or nitrate. EDTA prevented the interference of 50-fold of iron (II) or copper (II), 25-fold amounts of barium, iron (III), selenium (IV), tin (IV) or vanadium (V). Interferences of 25-fold of manganese (VII) or silver (I) have been completely removed by using oxalate as masking agent. A 25-fold of palladium (II) or Titanium (IV) has been completely removed by using EDTA or ascorbic acid as masking agent. However, for those ions whose tolerance limit has been studied their tolerance ratios are mentioned in Table 3.

## Applications

The present method was successfully applied to the determination of gold in a series of synthetic mixtures of various compositions (Table 4), and also in a number of real samples such as several standard reference materials (Table 5). The method was also extended to the determination of gold in a number of environmental, geological and soil samples. In view of the unknown composition of environmental water samples, the same equivalent portions of each sample were analyzed for gold content; the recoveries in both the “spiked” (added to the samples before the mineralization or dissolution) and the “unspiked” samples are in good agreement (Table 6). The results of biological and geological analyses by the spectrophotometric method were found to be in excellent agreement with those obtained by AAS (Table 7). The results of soil sample analysis by the spectrophotometric method are shown in Table 8.

### Determination of gold (III) in synthetic mixtures

Several synthetic mixtures of varying compositions containing gold (III) and diverse ions of known concentrations were determined by the present method using EDTA as a masking agent; and the results were found to be highly reproducible. The results are shown in Table 4. Accurate recoveries were achieved in all solutions.

### Determination of gold (III) in certified reference materials

A 0.5 g sample was accurately weighed and placed in a 50 mL Erlenmeyer flask following a method recommended by Parker (Parker, 1983). To this, 10 mL of concentrated HNO_3_ and 5 mL of concentrated HCl was added, carefully covering the flask with a watch glass until the brisk reaction subsided. The solution was heated and simmered gently after the addition of 5 mL of concentrated HNO_3_, until all carbides were decomposed. The solution was evaporated carefully to dense white fumes to drive off the oxides of nitrogen and then cooled to room temperature 25 ± 5 °C). After suitable dilution with de-ionized water, the contents of the Erlenmeyer flask were warmed to dissolve the soluble salts. The solution was then cooled and neutralized with a dilute NH_4_OH solution. The resulting solution was filtered, if necessary, through a Whatman No. 42 filter paper into a 25 mL calibrated flask. The residue was washed with a small volume of hot water and the volume was made up to the mark with de-ionized water.

A suitable aliquot of the above solution was taken in to 10 mL calibrated flask and gold content was determined by general procedure using EDTA as masking agent. The results are shown in Table 5. The results for total gold were in good agreement with certified values Table 5.

### Determination of gold (III) in environmental water samples

Each filtered (with Whatman No. 42) environmental water sample (100 mL) evaporated nearly to dryness with 10 mL of concentrated HNO_3_ in a fume cup-board and was heated with 10 mL of de-ionized water in order to dissolves the salts. The solution was then cooled and neutralized with dilute NH_4_OH solution. The resulting solution was then filtered and quantitatively transferred into a 25 mL calibrated flask and made up to the mark with de-ionized water.

An aliquot (1–2 mL) of this solution was pipetted into a 10 mL calibrated flask, and the gold content was determined as described under a procedure using EDTA as a masking agent. The analysis of environmental water samples from various sources for gold, and the results are given in Table 6.

### Determination of gold (III) in biological samples

Human blood (5–10 mL) or urine (10–20 mL) was collected in polyethane bottles from the affected persons. The samples were taken into a 100 mL micro-Kjeldahl flask. A glass bead and 10 mL of concentrated nitric acid were added and the flask was placed on the digester under gentle heating following a method recommended by Stahr (Stahr, 1991). When the initial brisk reaction was over, the solution was removed and cooled. Five milliliters of concentrated HNO_3_ was added carefully, followed by the addition of 0.5 mL of 70% HClO_4_, and heating was continued to dense white fumes, repeating HNO_3_ addition if necessary. Heating was continued for at least ½ h and then cooled. The content of the flask was filtered and neutralized with dilute ammonia. The resultant solution was then transferred quantitatively into a 10 mL calibrated flask and made up to the mark with de-ionized water.

A suitable aliquot (1–2 mL) of the final solution was pipetted out into a 10–mL calibrated flask, and the gold content was determine as described under Procedure using EDTA, ascorbic acid, tetra sodium pyrophosphate as a masking agent. The samples were also measured by atomic absorption spectrometry (Pyrzynska, 2005) for comparison of the results. The results of biological (human fluids) analyses by the spectrophotometric method were found to be in excellent agreement with those obtained by AAS. The results are given in Table 7.

### Determination of gold (III) in geological samples

An accurately weighed amount of 1 g sample was ground well and placed in 100 mL Erlenmeyer flask and dissolved in 15 mL of the acid mixture (aqua regia) was added, the solution was boiled on hot plate at 120 °C until the dissolution of the sample was completed, the resulting solution was carefully evaporated to small volume (2–3 mL) to remove the NO_3_− ions. After cooling and neutralized with dilute NH_4_OH solution. The resulting solution was then filtered and quantitatively transferred into a 20 mL calibrated flask and made up to the mark with de-ionized water.

An aliquot (1–2 mL) of this solution was pipetted into a 10 mL calibrated flask, and the gold content was determined as described under a general procedure using EDTA as a masking agent. The samples were also measured by atomic absorption spectrometry (Pyrzynska, 2005). The results of geological analyses by the spectrophotometric method were found to be in excellent agreement with those obtained by AAS. The results are given in Table 7.

### Determination of gold in soil samples

An air-dried homogenized soil sample (100 g) was weighed accurately and placed in a 100 mL micro-Kjeldahl flask. The sample was digested in the presence of oxidizing agent, following the method recommended by Jackson (Jackson, 1965). The content of the flask was filtered through a Whatman No. 42 filter paper into a 25 mL calibrated flask and neutralized with dilute NH_4_OH solution. It was then diluted up to the mark with de-ionized water.

Suitable aliquots (1–2 mL) were transferred into a 10 mL calibrated flask. The gold content was then determined, as described under general procedure using EDTA as a masking agent. The results are shown in Table 8.

## Conclusions

In the present work, a simple, sensitive, selective and inexpensive micellar method with the Au(III)-BSOPD complex was develop for the determination of gold in industrial, environmental, geological and soil samples. The presence of a micellar system (altered environment) avoids the previous steps of solvent extraction, and reduces the cost and toxicity while enhancing the sensitivity, selectivity and molar absorptivity. The molar absorptivities of the Gold-BSOPD complex formed in presence of the nonionic TX-100 surfactant are almost a 10 times higher than the value observed in the aqueous solution, resulting in an increase in the sensitivity and selectivity of the method. The apparent molar absorptivities were found to be 2.3 × 10^4^ L mol^−1^ cm^−1^ and 2.5 × 10^5^ L mol^−1^ cm^−1^ in aqueous and micellar media, respectively. Compared with other methods in the literature (Table 1), the proposed method has several remarkable analytical characteristics:

- The proposed method is very highly sensitive. The molar absorptivity of the complex is 2.5 × 10^5^ L mol^−1^ cm^−1^ and highest of all reagents in the literature (please see Table 1). Thus, amount of ng g^−1^ of gold can be determined without pre-concentration; its sensitivity corresponds with that of the graphite furnace atomic absorption method.
- The proposed method is highly selective. Because certified reference materials, environmental water and geological samples contain large amounts of metal ions and very low levels of gold, the selectivity of the determination of gold is most important.
- The proposed method is very simple, rapid and stable. The reaction of gold(III) with BSOPD is completed rapidly in micellar medium within 10 min at room temperature.

With suitable masking, the reaction can be made highly selective. The proposed method using BSOPD in the presence of aqueous micellar solutions not only is one of the most sensitive methods for the determination of gold, but also is excellent in terms of selectivity and simplicity. Therefore, this method will be successfully applied to the monitoring of trace amounts of gold, in real, environmental, biological, geological and soil samples.

## Disclosure

The authors report no conflicts of interest.

## Figures and Tables

**Table 1.** Review of reagents for spectrophotometric determination of gold.

**Reagent**	**λ_max_ (nm)**	**E (Lmol^−1^ cm^−1^)**	**Medium**	**Beer’s Law limits (mg/L)**	**Comments**	**Ref**
5-(2-Pyridyl) methylene-rhodanine	418	1.1 × 10^4^	HCl	–	1. Less-sensitive,	(Alfonso and Perez-Ruiz, 1981)
5-(6-Methylpyridyl) methylene-rhodanine	420	1.1 × 10^4^	HCl	–	2. Acetate buffer medium.	
					3. Less-Sensitive	
					4. pH dependent	
Morin	291	2.02 × 10^4^	HCl	0.2–12	1. Less—sensitive,	(Balcerzak et al. 2006)
					2. Indirect method,	
					3. UV range,	
					4. Temp: and time dependent.	
2-Carboxyl-1-naphthiorhodanine (CNTR)	540	1.35 × 10^5^	Dimethyl formamide (DMF) and EmuLsifier-OP (p-Octyl polyethylene glycol phenyl ether)	0.01–2.0	1. Solvent extractive so Lengthy and time consuming.	(Chen et al. 2006)
					2. DMF is highly toxic	
5 (2,4Dihydroxybenzylidine) rohodamine(DHBR)	558	8.45 × 10^4^	Cetylpyridinium bromide (CPB)	0.16–2.24	1. Less-sensitive	(El-Zawawy et al. 1995)
					2. Less-slective.	
					3. pH dependent.	
Thiamine and Phloxine	570	2.1 × 10^5^	Methylcellulose	0.02–0.8	1. pH, time and temp: dependent.	(Fujita et al. 1999)
2’-Aminoacetophenone isonicotinoyl hydrazone (2-AAINH)	440	3.50 × 10^4^	Aqueous Dimethyl formamide (DMF)	0.4–5.0	1. Less-sensitive,	(Gangadharappa et al. 2004)
					2. Organic medium.	
p-Sulfobenzylidene-thiorhodanine (SBDTR)	540	1.05 × 10^5^	HCl and emulsifier-OP	0.1–20	1. Sensitive	(Gao et al. 2005)
					2. SoLid Phase Extraction	
Propericiazine	511	3.85 × 10^4^	H_3_PO_4_	0.1–7.0	1. Less-sensitive,	(Gowda et al. 1984)
5-(2-hydroxy-5-nitrophenyla zo)thiorhodanine(HNATR)	520	1.37 × 10^5^	Emulsifier-OP (p-Octyl polyethylene glycol phenyl ether)	0.01–3.0	1. Solid Phase Extraction	(Hu et al. 2006)
					2. Less-sensitive	
Methylene Blue	657	1.08 × 10^5^	C_2_H_2_Cl_2_	0.04–1.58	1. Solvent extractive so lengthy and time consuming.	(Koh et al. 1986)
					2. pH dependent.	
Bromopyrogallol red	400	3.0 × 10^4^	–	0.1–3.0	1. Less-sensitive	(Matouskova et al. 1980)
					2. Strongly affected by ionic strength of solution	
Ethopazine hydrochloride(EPI)	513	2 × 10^4^	H_3_PO_4_	0.5–14.1	1. Less-sesitive,	(Melwanki et al. 2002)
Isopendyl hydrochloride(IPH)	512	2.1 × 10^4^	H_3_PO_4_	0.5–14.5	2. Complex stable only for 45 minutes.	
Tri-iso-octylamine	325	5.8 × 10^3^	CCl_4_	–	1. Less-sesitive,	(Mirza, 1980)
					2. Solvent extractive so lengthy and time consuming	
1,2,4,6-Tetraphenylpyridinium perchlorate	313	3.44 × 10^4^	HCl	0.05–0.5	1. Light-sensitive	(Ortuno et al. 1984)
					2. UV-range	
Photoinitiated gold sol	523	3.06 × 10^3^	Tritron X-100	0–150	1. Less-sensitive,	(Pal, 1999)
					2. Complex procedure,	
					3. Time dependent	
Amides and Amidines	320–400	0.06–1.2 × 10^4^	Chloroform or Benzene	–	1. Less-sesitive,	(Patell and Lieser, 1986)
					2. Solvent extraction	
5-(p-Aminobenzylidene)-thiorhodanine (ABTR)	550	1.23 × 10^5^	Emulsifier-OP(p-Octyl polyethylene glycol phenyl ether) and Dimethyl formamide (DMF)	0.01–3.0	1. Solid Phase Extraction with reversed phase polymer-based.	(Zhao et al. 2006)
					2. Complex stable for only 5 h.	
Bis (salicylaldehyde) orthophenylenediamine (BSOPD)	490	2.5 × 10^5^	TX-100	0.01–30	1. Ultrasensitive	Present work
					2. Highly selective	
					3. Aqueous reaction medium	
					4. Less toxic surfactant	

**Table 2.** Selected analytical parameters obtained with optimization experiments.

**Parameter**	**Selected value in aqueous solutions**	**Selected value in aqueous micellar solutions**
Wavelength, λ/nm	490	490
Acidity/M H_2_SO_4_	0.16–0.48 (preferably 0.4)	0.2–0.48 (preferably 0.2 M)
pH	0.48–1.23 (preferably 0.9)	0.83–1.2 (preferably 1.14)
Surfactant/10% TX-100/mL	–	1.0–3.5 (preferably 2)
Time/h	1 min–24 h (preferably 20 min)	1 min–24 h (preferably 5 min)
Temperature/°C	10–40 (preferably 25 ± 5)	10–40 (preferably 25 ± 5)
Reagent (fold molar excess, M:R)	1:10–1:20 (preferably 1:15)	1:10–1:25 (preferably 1:15)
Linear range/mg L^−^_l_	0.1–30	0.01–30
Molar absorption coefficient/L mol^−1^ cm^−^_1_	2.31 × 10^4^	2.52 × 10^5^
Sandell’s sensitivity/ngcm^−^_2_	60	5
Detection limit/μg L^−^_1_	15	1
Reproducibility (% R SD)*	1–5	0–2.5
Correlation coefficient (R^2^)	0.9959	0.9986

* Relative Standard Deviation (RSD).

**Table 3.** Table of tolerance limits of foreign ions^a^.

**Species x**	**Tolerance ratio^b^ x/Au^III^**	**Species x**	**Tolerance ration x/Au^III^**
Acetate	1000	Iron (II)	50^c^
Ascorbic Acid	75	Iron (III)	25^d^
Bicarbonate	100	Manganese (II)	100
Carbonate	500	Manganese (VII)	25^e^
Chloride	1000	Magnesium	100
Citrate	50	Molybdenum (VI)	100
Fluorides	100	Mercury (I)	100
EDTA	500	Mercury (II)	100
Nitrate	200	Neodymium(III)	100
Sulfate	1000	Nickel (II)	100
Oxalate	1000	Palladium(II)	25^d^
Phosphate	200	Potassium	100
Tartrate	200	Rhodium(III)	100
Ammonium(I)	100	Ruthenium(III)	100
Aluminum(III)	100	Selenium (IV)	25^d^
Arsenic (III)	100	Silver (I)	25^e^
Barium	25^d^	Sodium	100
Beryllium (II)	100	Strontium	50
Bismuth (III)	100	Thallium (I)	100
Cadmium	100	Tin(IV)	25^d^
Calcium	100	Titanium(IV)	25^f^
Chromium (III)	100	Tungsten (VI)	100
Cobalt (II and III)	100	Vanadium (V)	25^d^
Copper (II)	50^d^	Zirconium(IV)	100
Lanthanum(III)	100	Zinc	100
Lithium	100		

^a^Tolerance limit defined as ratio that causes less than 5% interference.

^b^Tolerance ratio, [Species (x)]/Au^III^ (w/w).

^c^With 50 mg L^−1^ EDTA.

^d^With 500 mg L^−1^ EDTA.

^e^With 500 mg L^−1^ Oxalate.

^f^With 50mg L^−1^ Ascorbic acid.

**Table 4.** Determination of gold in some synthetic mixtures.

**Sample**	**Composition of mixture/mg L^−1^**	**Gold(III)/mgL^−1^**		**Recovery ± s (%)**
		**Added**	**Found^a^**	
A	Au^III^	0.5	0.49	98 ± 0.5
		1.00	1.00	100 ± 0.0
B	As in A + Na (25) + Be^2+^ (25)	0.5	0.50	100 ± 0.0
		1.00	0.99	99 ± 0.3
C	As in B + Zn (25) + Co (25) + EDTA(50)	0.50	0.49	98 ± 0.5
		1.00	0.99	99 ± 0.2
D	As in C + Ca(25) + Cr^3+^(25)	0.5	0.50	102 ± 0.6
		1.00	1.02	102 ± 0.4
E	As in D + Mn^2+^(25) + Ag^+^(10)	0.5	0.52	104 ± 1.2
		1.00	1.06	106 ± 1.0
F	As in E + Ni^2+^(25) + Hg^2+^(25)	0.5	0.54	108 ± 1.5
		1.00	1.09	109 ± 1.2

^a^Average of five analyses of each sample.

**Table 5.** Determination of gold in certified reference materials.

Certified reference materials* (Composition)	**Gold (mg L^−1^)**		**Relative error (%)**
	**Certified value**	**Found (n = 5)**	
OXG 60 (SiO_2_, Al_2_O_3_, Na_2_O, K_2_O, CaO, MgO, TiO_2_, MnO, P_2_O_5_, Fe_2_O_3_, LOI)	1.025	1.015	1.0
OXG56 (SiO_2_, Al_2_O_3_, Na_2_O, K_2_O, CaO, MgO, TiO_2_, MnO, P_2_O_5_, Fe_2_O_3_, LOI)	0.611	0.605	0.6
SH 24 (SiO_2_, Al_2_O_3_, Na_2_O, K_2_O, CaO, MgO, TiO_2_, MnO, P_2_O_5_, Fe, S)	1.326	1.315	1.1

* These CRMs obtained from Rock labs Ltd., Auckland, New Zealand.

**Table 6.** Determination of gold in some environmental water samples.

**Sample**		**Gold/μg L^−1^**		**Recovery ± s (%)**	**s_r_^b^ (%)**
		**Added**	**Found^a^**		
Tap water		0	5.0		
		100	104.0	99 ± 0.3	0.45
		500	505.0	100 ± 0.0	0.00
Well water		0	8.5		
		100	109.0	100.4 ± 0.5	0.32
		500	512.0	100.6 ± 0.2	0.35
Rain water		0	0.0		
		100	100.5	100.5 ± 0.2	0.24
		500	500	500 ± 0.0	0.00
River water	Lake^c^ (upper)	0	25.0		
		100	123.0	98 ± 0.3	0.19
		500	520.0	99 ± 0.2	0.25
	Lake^c^ (lower)	0	22.0		
		100	120.0	98 ± 0.5	0.30
		500	522.0	100 ± 0.0	0.00
	Indus (upper)	0	12.8		
		100	112.0	99 ± 0.5	0.35
		500	513.0	100.0 ± 0.2	0.19
	Indus (lower)	0	9.5		
		100	110.0	100.5 ± 1.0	0.35
		500	512.0	100.5 ± 0.8	0.21
Sea water	Arabian Sea (upper)	0	4.5		
		100	105	100.5 ± 1.0	0.35
		500	506	100.3 ± 0.7	0.33
	Arabian Sea (lower)	0	5.5		
		100	106	100.5 ± 0.2	0.35
		500	506	100.1 ± 0.3	0.31
	Jewels wastewater^d^	0	125.0		
		100	225.0	100.0 ± 0.0	0.00
		500	528.0	100.5 ± 0.4	0.28
Drain water	Aral wah^e^	0	45.0		
		100	148.0	102 ± 1.0	0.24
		500	545.0	100 ± 0.0	0.00
	Fiber tex^f^	0	25.0		
		100	127.5	102 ± 0.8	0.49
		500	530.0	100.9 ± 0.3	0.35
	Jhampur^g^	0	28.5		
		100	130.0	101 ± 0.9	0.15
		500	525.0	99 ± 0.5	0.26

^a^Average of five replicate determinations.

^b^The measure of precision is the relative deviation(sr).

^c^The Manchar Lake, Dadu, Sindh.

^d^Jewelers shops from Khairpur, Karachi and Hyderabad, Sindh.

^e^Aral wah, Dadu, Sindh.

^f^Kotri, Hyderabad.

^g^Kotri, Hyderabad.

**Table 7.** Concentration of gold in biological (blood and urine) and geological samples.

		**Gold/μgL^−1^**					
**Serial No**	**Sample**	**AAS^a^** **(n = 5)**		**Proposed method** **(n = 5)**		**Relative error%**	**Sample Source**
		**Found**	**RSD^b^%**	**Found**	**RSD%**		
1	Blood	55.2	1.0	56.5	1.0	2.3	Normal adult (Male)
	Urine	13.8	1.2	14.6	1.3	1.4	
2	Blood	423.2	1.5	425.5	1.2	0.5	Jeweler’s blood(Male)^c^
	Urine	98.5	1.8	99.8	1.8	1.3	(Hyderabad)
3	Blood	128.8	1.3	127.5	1.5	1.0	Jeweler’s blood(Male)^c^
	Urine	32.5	1.8	31.8	2.0	1.6	(Tandojam)
4	Rock_1_^e^	15.8	1.2	16.3	1.0	3.0	MTL^d^
							Peshawer
5	Rock_2_^e^	16.7	1.5	17.2	1.2	2.9	Karak Mountain
6	Rock_3_^e^	15.1	1.3	15.5	1.5	2.5	Baka Khail Mountain

a Atomic Absorption Spectrophotometry.

b Relative Standard Deviation.

c Samples were from Jewelers of Hyderabad.

d Mineral Testing Laboratory, 164-C, Industrial Estate, Jamrud Road, Peshawar.

e Value in ng g^−^1.

**Table 8.** Determination of Gold in some surface soil samples.

**Serial No**	**aGold/ng g^−1^**	**Sample source**
S_1_	10.2 ± 0.5^b^	Traffic soil (Hyderabad bus terminal)
S_2_	8.5 ± 0.8	Roadside soil (Hyderabad— Karachi highway)
S_3_	6.8 ± 0.7	Marine soil (Sand of Arabian Sea)
S_4_	20.5 ± 1.0	Industrial Soil (Pharmaceutical Company)
S_5_	7.5 ± 0.8	Agricultural soil (Sindh University Campus)

^a^Average of five analyses of each sample.

^b^The measure of precision is the standard deviation, ± s.
